# Gallstone Ileus: A Rare Incidental Gallstone Migration Seen on CT in a Patient With Traumatic Splenic Laceration

**DOI:** 10.7759/cureus.83074

**Published:** 2025-04-27

**Authors:** Vanessa Lin, Kyeong Ri Yu, Chonn J Cadiz, Lindsey C Ferro

**Affiliations:** 1 Department of Surgery, Virginia Commonwealth University Health System, Richmond, USA; 2 Department of Surgery, Richmond Veterans Affairs (VA) Medical Center, Richmond, USA

**Keywords:** cholecystectomy, digestive system fistula, gallstone ileus, incidental finding, intestinal obstruction

## Abstract

Gallstone ileus, a rare but severe complication of cholelithiasis, causes bowel obstruction and is typically associated with high morbidity and mortality. Here, we present the case of a 76-year-old male who initially presented with left upper quadrant abdominal pain after a ground-level fall, with the incidental finding of cholelithiasis. Over subsequent days, the patient developed symptoms of small bowel obstruction, and further CT imaging showed a new-onset gallstone ileus. Exploratory laparotomy confirmed the obstruction within the distal jejunum, and a large gallstone was successfully extracted. This case highlights the importance of considering gallstone ileus in patients with atypical presentation and underscores the potentially critical role of CT imaging in diagnosing and tracking gallstone movement. Increased awareness and understanding among clinicians, particularly in trauma settings, are essential for timely intervention and improved patient outcomes.

## Introduction

Gallstone ileus is a rare but serious complication of cholelithiasis, causing bowel obstruction when a gallstone enters the gastrointestinal (GI) tract. It accounts for approximately 1% to 4% of all cases of mechanical small bowel obstruction and is more commonly seen in elderly patients. Incidence is 30 to 35 cases per million admissions; however, despite its rarity, it is associated with high morbidity and mortality (in-hospital mortality of 6% to 13%) due to delayed diagnosis, the advanced age and frailty of most affected patients, and the risk of complications such as bowel perforation, peritonitis, and sepsis [1]. 

Gallstone migration usually follows acute cholecystitis, where localized inflammation leads to fistula formation through the gallbladder wall, commonly to the duodenum. While the duodenum is the most frequent site, fistulas to the colon or stomach have also been reported [2]. Once a fistula is established, gallstones can migrate into the GI tract, and those larger than 2 cm to 2.5 cm are more likely to cause obstruction, most commonly at the ileocecal valve due to its narrow lumen [2]. Imaging, especially CT, is crucial for diagnosis. Rigler's triad (intestinal obstruction, pneumobilia, and an ectopic gallstone) is diagnostic but variably reported on abdominal X-ray (ranging from 40% to 70%) [3-6]. Treatment is dependent on clinical presentation, but enterolithotomy is generally preferred, as medical management alone is associated with a higher mortality rate [3,7].

Rarely, gallstone ileus can be precipitated by trauma, as presented in our following case, where a ground-level fall preceded the development of gallstone ileus seven days later. We hypothesize that sudden increases in intra-abdominal pressure following blunt trauma may accelerate gallstone migration through an existing cholecystoenteric fistula, hastening the onset of obstruction. The following case highlights this uncommon presentation, emphasizing the need for a high index of suspicion in patients with a history of both trauma and gallstone disease.

## Case presentation

**Figure 1:**
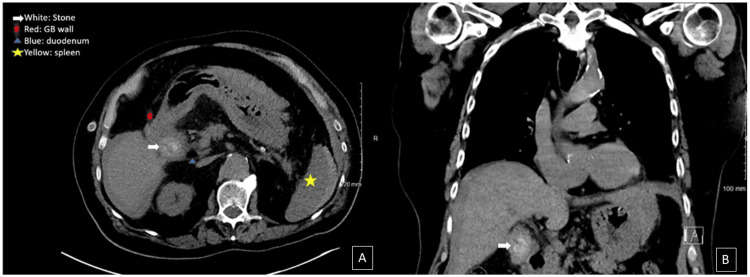
CT thorax on day one of hospitalization A: Transverse section shows a mass (white arrow) that is notably present within the gallbladder (red dot); B: Coronal section showing the mass (white arrow) notably present within the gallbladder

**Figure 2:**
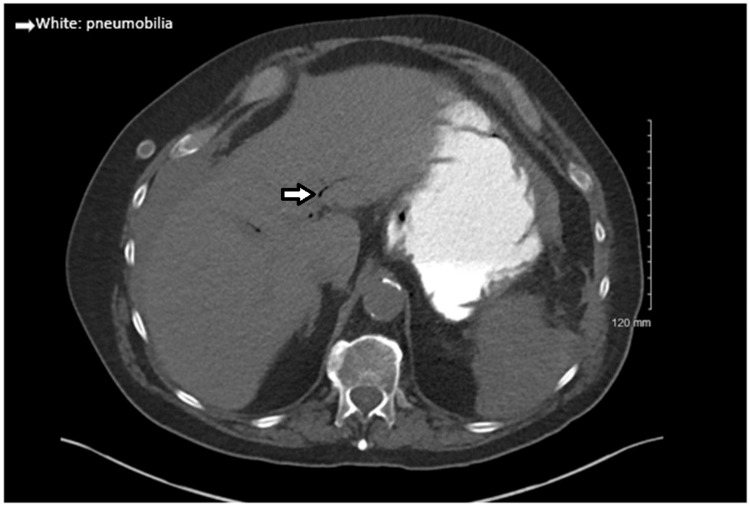
Transverse section of CT abdomen on day three of hospitalization shows pneumobilia without gallstone present (white arrow)

We present the case of a 76-year-old male who initially presented with splenic laceration and hemoperitoneum four days after a ground-level fall while on apixaban (Eliquis). Initial physical examination revealed left upper quadrant abdominal tenderness with guarding. A CT of the chest, abdomen, and pelvis found a grade II splenic laceration and hemoperitoneum. Incidentally, a gallstone was also noted to be present within the gallbladder in transverse and coronal views (Figure 1). Though he was hemodynamically stable, he was admitted to the general surgery service for observation due to imaging findings and the recent use of apixaban. Serial abdominal exams and labs demonstrated a mild decrease in hemoglobin levels, within 1-2 g/dL, over two days, and the patient remained hemodynamically stable. On day three of hospitalization, the patient developed new-onset nausea with vomiting. His physical exam was concerning for small bowel obstruction, and subsequent nasogastric tube placement yielded one liter of gastric and bilious output. A repeat CT revealed a stable grade II splenic laceration and diffusely dilated small bowel, with intrahepatic pneumobilia and new common bile duct dilation (Figure 2).

**Figure 3:**
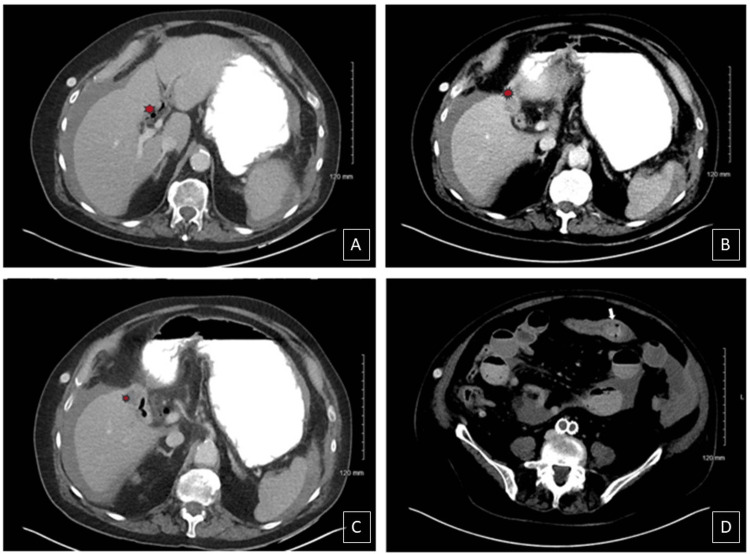
Transverse sections of CT abdomen with contrast on day three of hospitalization A-C: Gallbladder without stone and intraluminal air present (red dot); D: Stone present within the jejunum, distal to the initial position observed in Figure 1 (white arrow)

**Figure 4:**
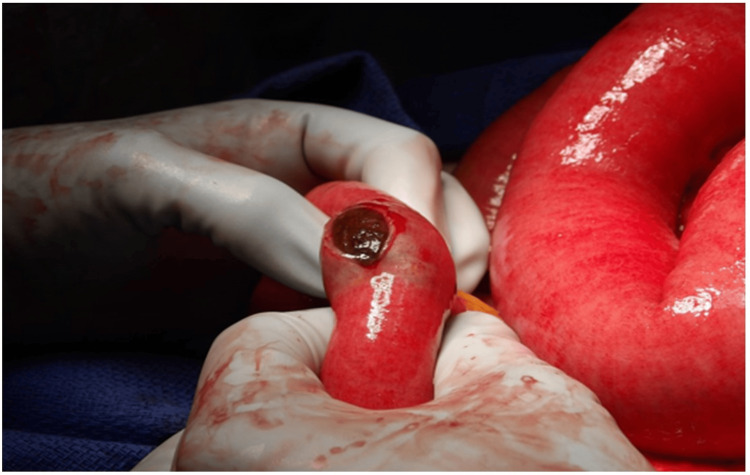
Intraoperative image of obstruction in the distal jejunum The enterotomy was made on the small bowel directly overlying the mass, revealing a likely gallstone. Bowel coloration and perfusion were initially dusky, but improved after removal of the stone.

**Figure 5:**
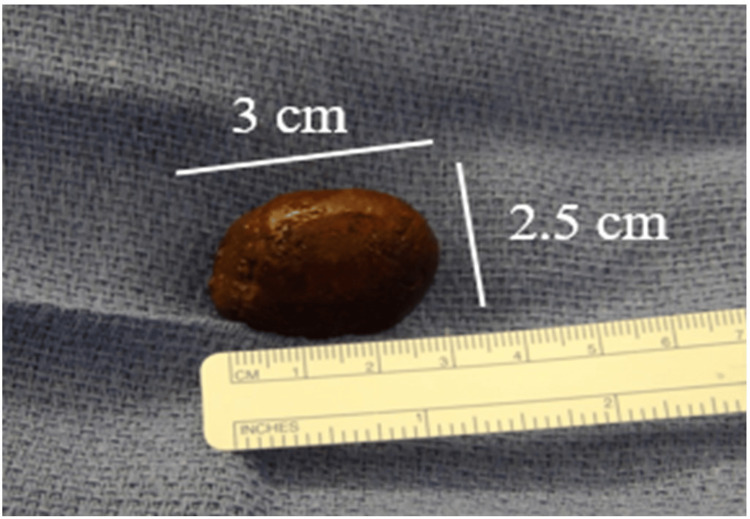
Gross appearance of bowel content (gallstone) The specimen was sent to pathology for gross confirmation; no microscopic sections were taken. It measured 3 x 2.5 x 2 cm.

Gallstone migration was observed to the jejunum, with surrounding dilated small bowel and fecal content on imaging (Figure 3). With clinical presentation and imaging concerning for gallstone ileus, exploratory open laparotomy was performed. We noted that initially the small bowel appeared dusky with an obstruction in the distal jejunum (Figure 4). A 3 x 2.5 x 2 cm gallstone was extracted from the jejunum (Figure 5). Bowel perfusion improved in coloration after removal of the gallstone, and no resection was necessary. Given the presence of a likely chole-duodenal fistula, a cholecystectomy was not completed during this perioperative course. Splenectomy was performed due to hemoperitoneum and ongoing bleeding observed from a hilar capsular laceration. With no other lesions or abnormalities found, the abdomen was closed, and the patient was transferred to the surgical ICU for further care. His postoperative course was unremarkable, and he was eventually discharged home on postoperative day six. He was found to be recovering well at his two-week postoperative appointment.

## Discussion

Gallstone ileus, a rare presentation of small bowel obstruction, is most commonly reported as a sequela of recent acute cholecystitis. Here, we described the case of a 76-year-old male patient who developed symptoms of gallstone ileus approximately seven days after a ground-level fall in the setting of splenic laceration and hemoperitoneum while on apixaban. Interestingly, a gallstone was found incidentally in the gallbladder on initial imaging without symptoms of acute cholecystitis. With interval imaging three days later, the same gallstone was seen within the jejunum, indicating a route of travel likely through a bilio-enteric fistula or a dilated common bile duct. This finding correlates with developing clinical symptoms of small bowel obstruction.

In available literature, gallstone ileus after trauma has only been reported in one case in a woman with previous episodes of acute cholecystitis, which likely led to fistula formation [8]. Our patient did not have a history of cholecystitis or biliary colic symptoms. Trauma associations may be underrepresented in literature simply due to the low incidence of gallstone ileus; however, pathophysiology could be contributory. Chronic recurrent cholecystitis leads to erosion through the gallbladder wall from the pressure effects of the gallstone. Transformation from sites of erosion to fistula formation to the adjacent gastrointestinal tract creates a pathway for gallstones to pass through, leading to earlier detection and intervention regularly. Although less common, the common bile duct or a dilated papilla of Vater can also serve as sites for gallstone entry [2]. Additionally, chronic recurrent cholecystitis increases the probability of earlier surgical intervention with cholecystectomy, which is preventative for gallstone ileus. 

The role of CT imaging in literature, while well established in diagnosing gallstone ileus, has not been well reported in showing the movement of gallstones through the GI tract. We propose that several factors likely contribute to this, including the intermittent nature of bowel obstruction, variability in gallstone size, and the potential for rapid transit through the digestive tract. Additionally, signs of bowel obstruction and physiologic materials present in the bowel may further complicate the direct visualization of gallstones.

When reviewing the workup and diagnosis of gallstone ileus, patient factors and community resources available are also important considerations. The diagnosis of gallstone ileus often involves a combination of clinical evaluation and imaging studies, with radiological examinations such as plain abdominal X-rays, ultrasound, CT, and occasionally endoscopic retrograde cholangiopancreatography (ERCP). Studies in the past have underscored the significance of imaging techniques in accurately diagnosing gallstone ileus [4]. Serial CT scans are infrequently completed for uncomplicated ileus because of the higher monetary and resource costs as compared to other modalities, such as plain abdominal film series, which is the standard of care for imaging of uncomplicated ileus. Gallstone ileus, however, is the result of a mechanical obstructive process in which CT imaging offers comparatively higher accuracy [9]. Thus, an increased awareness among providers-in-training of this misnomer is important to avoid misdiagnosis. Primary criteria for diagnosis on CT include detection of small bowel obstruction with one of the following: ectopic gallstone; abnormal gallbladder with air collection, air-fluid level, or fluid accumulation with irregular walls; bowel loop dilation; bilio-enteric fistula; pneumobilia; or extraluminal fluid. Notably, the most common CT correlations are small bowel obstruction, ectopic gallstone with rim or total calcification, and abnormal gallbladder with intraluminal air [5,6]. 

Consequently, while CT may reveal signs of bowel obstruction and the presence of gallstones, witnessing the passage in time of a gallstone through the small bowel on imaging is an uncommon occurrence. Further investigation is needed to evaluate whether serial CT imaging facilitates earlier diagnosis of small bowel obstruction as opposed to clinical evaluation, which may be more biased in interpretation. 

Lastly, surgical management of gallstone ileus remains controversial. Debated options include enterolithotomy alone, a one-stage procedure that includes cholecystectomy and fistula closure, and the two-stage procedure with interval cholecystectomy. As the typical population affected by gallstone ileus tends to be higher in age and the number of comorbidities at the time of presentation, the consensus is that neither an urgent nor delayed cholecystectomy is necessary and can only add to the risks of complications and mortality [10]. As our patient was an elderly patient with traumatic hemoperitoneum on previous anticoagulation requiring a concomitant splenectomy, we also elected to forgo cholecystectomy.

## Conclusions

Gallstone ileus, although a rare form of small bowel obstruction, presents unique diagnostic and therapeutic challenges, particularly when it arises without preceding acute cholecystitis symptoms, as demonstrated in the case of our 76-year-old patient. The occurrence following trauma and the incidental findings highlight the importance of considering gallstone ileus in differential diagnoses, even in atypical presentations. The CT imaging improves not only the identification of gallstones but also the tracking of their movement through the gastrointestinal tract, despite challenges posed by variable obstruction patterns and gallstone characteristics. Increased awareness and understanding among clinicians are essential to improve diagnosis and management, underscoring the need for further research into the benefits of serial CT imaging versus traditional clinical evaluation. This case also suggests a potential underrepresentation of trauma-associated gallstone ileus in the literature, warranting further exploration of its pathophysiology and incidence. Enhanced diagnostic vigilance and resource allocation, particularly in trauma settings, could lead to timely intervention and improved patient outcomes in gallstone ileus cases.
